# A free customizable tool for easy integration of microfluidics and smartphones

**DOI:** 10.1038/s41598-022-13099-z

**Published:** 2022-05-27

**Authors:** Federico Schaumburg, Juan P. Vidocevich, Gabriel S. Gerlero, Nazarena Pujato, Joana Macagno, Pablo A. Kler, Claudio L. A. Berli

**Affiliations:** 1 Instituto de Desarrollo Tecnológico para la Industria Química (INTEC, UNL-CONICET), Colectora RN 168, S3000GLN Santa Fe, Argentina; 2 Centro de Investigación de Métodos Computacionales (CIMEC, UNL-CONICET), Colectora RN 168, S3000GLN Santa Fe, Argentina; 3 Laboratorio de Tecnología Inmunológica (FBCB, UNL), Colectora RN 168, S3000GLN Santa Fe, Argentina

**Keywords:** Immunochemistry, Computational platforms and environments, Databases, Image processing, Software, Diagnostic markers, Diagnosis, Biomedical engineering, Software, Fluid dynamics, Techniques and instrumentation

## Abstract

The integration of smartphones and microfluidics is nowadays the best possible route to achieve effective point-of-need testing (PONT), a concept increasingly demanded in the fields of human health, agriculture, food safety, and environmental monitoring. Nevertheless, efforts are still required to integrally seize all the advantages of smartphones, as well as to share the developments in easily adoptable formats. For this purpose, here we present the free platform *appuente* that was designed for the easy integration of microfluidic chips, smartphones, and the cloud. It includes a mobile app for end users, which provides chip identification and tracking, guidance and control, processing, smart-imaging, result reporting and cloud and Internet of Things (IoT) integration. The platform also includes a web app for PONT developers, to easily customize their mobile apps and manage the data of administered tests. Three application examples were used to validate *appuente*: a dummy grayscale detector that mimics quantitative colorimetric tests, a root elongation assay for pesticide toxicity assessment, and a lateral flow immunoassay for leptospirosis detection. The platform openly offers fast prototyping of smartphone apps to the wide community of lab-on-a-chip developers, and also serves as a friendly framework for new techniques, IoT integration and further capabilities. Exploiting these advantages will certainly help to enlarge the use of PONT with real-time connectivity in the near future.

## Introduction

Internet-of-Things (IoT) emerged in the last few years and rapidly grew to become one of the most important high-tech trends^1^. Although there is no clear consensus on IoT definition, it may be thought as the interconnection of people, objects and systems for smart data collection, processing and reaction^2^. IoT opens limitless possibilities in applications including agriculture, smart cities, commerce and industry^3^. Most authors identify three main layers within IoT: a first layer for sensing purposes; a second layer in charge of communication, storage, and processing; and a third layer to interface the user, providing information management and data analytics^4^. The second and third layers are transverse to all application fields, and have shown great advances in the last years^2^. In contrast, the first layer is application-specific and its readiness level is then heterogeneous. A relevant case is the detection of biomolecules in real time, which is connected to the concept of point-of-need testing (PONT), i.e. in-field, real-time chemical determinations. When the test is related to human health, this concept is referred to as point-of-care testing (POCT).

PONT is demanded in the fields of human and animal health, food safety, environmental monitoring and surveillance^5^. In 2003, the World Health Organization defined the ASSURED criteria to define the ideal POCT, which should be affordable, sensitive, specific, user-friendly, rapid and robust, equipment-free and deliverable to end users^6^. This concept was recently reaffirmed and renewed, with the REASSURED acronym, now including real-time connectivity and easy specimen collection^7^. Even though many efforts have been conducted from academic and industrial sectors, the PONT necessity is still unmet^8^, excluding some exceptional cases^9^. The progress achieved on PONT is based on microfluidic technology, in any of its forms: glass chips^10^ lateral flow immunoassays (LFIA)^11^, paper-based analytical devices (µPAD)^12^, polymeric microfluidic devices^13^, or hybrid devices^14^. Microfluidics presents inherent advantageous characteristics like requiring small amounts of sample and reagents, decreased reaction times^15^, and the potential to implement chemical operations on a reduced chip^16^. However, most of the approaches fail to accomplish all REASSURED criteria simultaneously, mainly because additional hardware is needed for the purpose of providing off-chip operations like heating, pumping, valve actuation and reading. In the best case, all the operations needed are centralized in a single piece of equipment^17,18^. Usually, sensitivity, specificity and speed are addressed but neglecting autonomy, portability, deliverability and sometimes cost.

In the last decade, the integration of smartphones and microfluidic devices has been proposed^19^ and explored in many directions, as described in a series of recently published review articles^20–22^. Smartphones offer many complementary advantages, like computing power, data storage, power sourcing, connectivity, a diversity of sensors, traceability, geolocation, the possibility of guiding the user throughout the test^23,24^, as well as control of the validity and quality of measurements, using specific apps (hereafter *app* will be used to mean *software application*, while *application* will be used to denote a *specific field of use*). Even more, smartphones are a natural gateway for PONT to give place to complex IoT applications involving sensing, smart decision making and even actuation. The majority of the smartphone-based approaches also rely on additional hardware. According to the application, this hardware can be: (i) a potentiostat^25,26^, (ii) a dark-box for digital imaging, meant to provide proper geometry and light parameters^26–28^, (iii) a heating module^29,30^, (iv) a fluid handling module or (v) combinations of the previous ones^17,31–34^. The accessories create additional costs, may present hygiene issues, and usually are phone-model specific^35^. These approaches are also detrimental to portability, autonomy and deliverability, deviating from the ASSURED criteria.

Interestingly, some PONT microfluidic devices use smartphones without additional hardware. This approach mainly relies on digital imaging using the integrated camera, with rare exceptions^36^. Nowadays smartphones are ubiquitous^37^ and PONT approaches could include them without loss of portability, deliverability or autonomy. Moreover, smartphones can be used to improve other characteristics of ASSURED, like sensibility, specificity, usability and robustness. Even more, smartphones guarantee the real time connectivity deemed necessary in REASSURED. Advantages of only using smartphones compared to the approaches using additional hardware are: (i) more autonomy and portability, (ii) lower cost, (iii) easier handling, (iv) fewer cross-contamination and hygiene issues^26^. When solely using a smartphone, different reproducibility issues emerge due to: (i) ambient light intensity and color variability^38–41^, (ii) non-uniform illumination^41^, (iii) variability in geometric parameters like capture distance and angles^41^, (iv) variability in camera parameters (focus mode, exposure time, ISO, resolution)^41,42^, (v) phone-specific pre-processing algorithms^41,43^ and (vi) brand/model-specific variability^40^. Various strategies have been developed to circumvent these issues. For example, chromatic and light source effects can be separated by using different color spaces^35,39,40,42,43^ or advanced processing methods^41^. The built-in LED can be used as the main light source to address light uniformity^35,42^. Also, on-site or on-chip calibration can be performed. The former^40^ requires additional time, testing, and training, while the latter involves additional features in the chip, such as color^35,39^ or chart references^41,43^, controls^44,45^, or standard addition assays^38^. Also, alignment marks^41,42,46^ or frames^47^ can be included to control the variability of geometric parameters.

Irrespective of the inclusion of additional hardware, most of the works exploit a limited set of features offered by phones, without taking advantage of capabilities such as image processing, timers, alarms, and traceability controls^48^, or the aforementioned ability to guide and control the user actions through step-by-step multimedia instructions^49^. Moreover, previous works are neither customizable, free or open-source, meaning that developed tools cannot be easily adopted by others, hindering fast prototyping and technology accessibility. The few exceptions to some of these issues are an open-source potentiostat for electrochemistry-related applications^26^, a free stand-alone software for colorimetry analysis for test developers^50^, and a paid software product where customization is undertaken by a team of programmers^51^. Thus, to leverage IoT-PONT applications, a platform enabling easy integration of smartphones to microfluidic-based projects is needed.

This paper reports the development of a free platform called *appuente*, for the easy integration of microfluidic chips, smartphones, and the cloud, which can be further exploited for IoT. This platform includes a web app for PONT developers, to easily customize and manage the data of the administered tests. It also includes a smartphone app, to guide and control the end-user actions throughout the test, by using tools and peripherals provided by smartphones. The platform was conceived from the beginning to be customizable and ideally serve any application, being also flexible to add further features. Moreover, *appuente* is meant to help test developers to take advantage of all the potential offered by smartphones, like (i) step-by-step multimedia guidance, (ii) control mechanisms like timers, alarms and tracking, (iii) compatibility with all the mentioned strategies to improve reproducibility and (iv) cloud connectivity and IoT integration, among others. Although *appuente* is mainly thought for the integration of microfluidics and smartphones without additional hardware, the platform is also compatible with several miniaturized laboratory assays (see for example^52^), as well as with applications that involve additional hardware (see for example^53,54^). In order to illustrate and validate the performance of the platform, three application cases are presented. The first one is a dummy application to detect levels of gray, (mimicking e.g. the output of quantitative colorimetric test), which enables full characterization of the system. The second application case is a milli-fluidic root elongation assay for pesticide toxicity assessment^52^. The last case is a LFIA for leptospirosis detection^55^. The potential of the platform to produce equipment-free, robust, qualitative and quantitative assays is achieved in this article by including proper calibration features within the chip. Finally, it is worth to remark that we are presenting a mixed platform that gathers all the advantages that mobile devices and the cloud offer to microfluidic devices for PONT. Some of these advantages have been previously reported, but they were given separately and some of them are inaccessible. Here, we integrate all the improvements on *appuente* and provide them in an easily accessible format.

## Appuente

Before going through a detailed description of the platform, it is relevant to introduce some declarations. Firstly, it is worth mentioning that *puente* means *bridge* in Spanish, hence the name *appuente* sets the mission of making accessible the large number of smartphones capabilities to the community of microfluidic developers. Secondly, it should be noted that there are three key roles in *appuente*: *developers*, *users* and *patients*. The first one is the person/team developing the test, who will mainly use the web app. The *user* is the person administering the test, like a health-care worker, in the case of POCT requiring trained personnel, or the interested itself, in the cases of elementary tests. The *user* will handle the mobile app. The *patient* role, which may exist or not depending on the application, is the person to whom the test is administered. This role might only provide the sample to be analyzed or, in the case of completely autonomous PONTs, can be simultaneously the *user*. Finally, it must be stated that *appuente*, in its present form, is intended for research, education, and product prototyping. The platform should not be straightforwardly used in operational environments that require compliance with safety policies or other regulation. In what follows, we describe the main components of the developed platform: microfluidic chip, mobile app, and web app.

### Microfluidic chip

**Figure 1:**
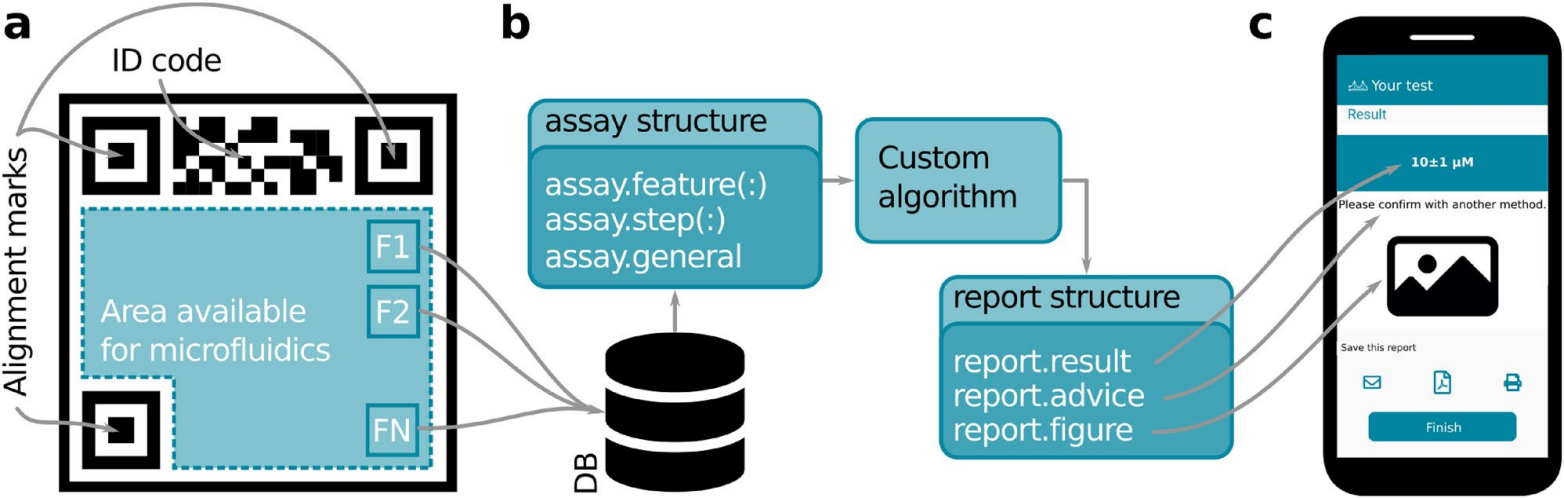
Elementary parts of *appuente*. (**a**) Microfluidic chip structures required by *appuente*. Besides alignment marks and ID code, the developer must define the features that will be used to obtain the test result. In this example, N features (F1, F2,...FN) were defined. (**b**) Inputs and outputs for the custom algorithm. The developer must provide a .m file with the desired algorithm. This algorithm will receive a data structure with the available information about the project and the particular assay, and must return another structure to be used in the final report. (**c**) The information returned is shown in the smartphone, at the end of the process, in a customized report.

As represented in Fig. 1a, *appuente* requires microfluidic chips to include alignment marks and an ID code. The marks are meant to avoid the geometric variability from assay to assay, due to different distances and angles when the image is taken. The ID code is meant to identify the test type and the singular chip, in order to apply the controls described in the next subsection. Also, the *features* that determine the assay result must be defined. The number, size, position, and meaning of such features depend on the application, and will be usually associated with a calibration procedure and/or result reading (for example, test and control lines in LFIA).

The ID codes and alignment marks are automatically generated by the platform and provided in a vector graphics file (SVG or PDF format) together with the mentioned features. The developer can use such file as a starting point to add the application-specific microfluidics, and then to materialize the chip (via wax printing, 3D printing, laser engraving). Note that *appuente* does not require a particular material for the chip, which depends entirely on the design purposes of the developer.

### Mobile app

**Figure 2:**
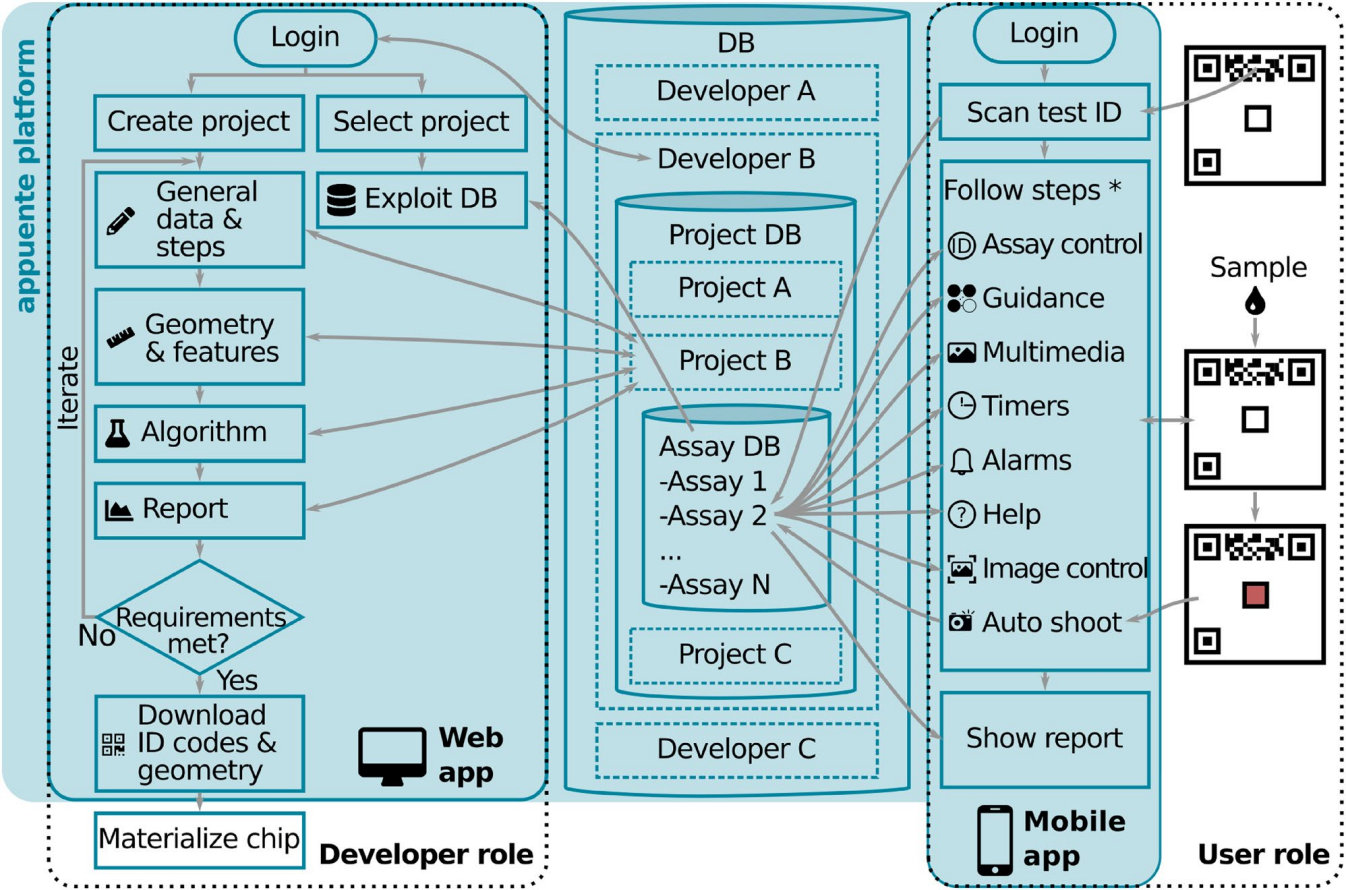
Representation of the typical flow diagram followed by *appuente* developers and users and interactions through the database (DB). (*) Note that after the ID code scan, the specific steps to be followed depend on the project; thus, the “Follow steps” box enumerates a subset of the offered possibilities.

As represented in Fig. 2, the operation of the mobile app is as follows. First, the user scans the ID code of the chip using a scanner built-in the app. Using this ID code the mobile app downloads the test instructions from the server. At this point, the server runs several controls to check for expiration date, no-reuse (for disposable tests), and batch validity (for possible device recalls). If the chip passes such controls, the app guides the user throughout the test-specific steps using the multimedia resources provided by the developer, such as text and images. During this stage, user actions can be *controlled* by the app with alarms and timers, e.g. by setting a window of opportunity for reading results. In the last step, the mobile app helps the user to take a picture with standard characteristics (regarding geometry and ambient light conditions), in order to guarantee result reproducibility within a range. After this operation, the app sends the cropped features inside an *assay* data structure to the server, together with other relevant information about the test. The server runs a custom algorithm in GNU Octave and sends back a *report* structure (refer to the Supplementary Information for detail about the named structures) to the app. This process is schematized in Fig. 1b. Finally, the mobile app shows the results to the user (Fig. 1c), along with other information deemed necessary by the developer (e.g. error ranges and recommendations). A video showing the operation of the mobile app is provided with the Supplementary Information.

### Web app

The web app (*appuente.com*) is meant for test developers and allows guided customization of both the mobile app and the microfluidic chip, to cover the application needs. The developer can follow two workflows, as represented in Fig. 2: one for project creation and the other for data management. In the former, the developer firstly logs in and creates a new project, then fills a form with general information about the test (e.g. name, description, icon) and specific information that will be part of the mobile app: e.g test summary and number, type and details of steps to be followed. Note that the last step will always be a smart picture, i.e., a picture where geometric and ambient-light parameters are controlled. Then, the developer loads geometric information about the chip and its features. Next, uploads the algorithm that will be applied to the information collected in the smart picture step. Such algorithm has to be uploaded in a .m file, and will be run in GNU Octave, a free, open-source alternative to Matlab and compatible with it. This algorithm has to be created and debugged by the developer, and the only requirement is that the input (*assay*) and output (*report*) data structures are used for communication with the platform. Some of the algorithms used in this work are given as examples in the Supplementary Information. Next, the developer has to design the report that will be seen by the user. Finally, the developer can download the vector geometry of the chip to add the microfluidics and materialize (e.g. by printing or engraving) the actual chips. The developer is expected to iterate this workflow until the mobile application behaves as desired. Note that, if the developer wishes further customization (e.g. to serve specific normative or to manage the database in another server), the project can be *ejected* from the platform. With this action, the source code of the mobile app is provided under a BSD-3-Clause License and can be downloaded to be freely managed elsewhere.

In the second workflow, where the test is already designed and available to users, the developer can access the database and manage the collected information. In the current version, *appuente* only allows data inspection and downloading; on-site analytics and integration to external databases will be included in the near future.

## Results

### Application case #1: Grayscale detector

**Figure 3:**
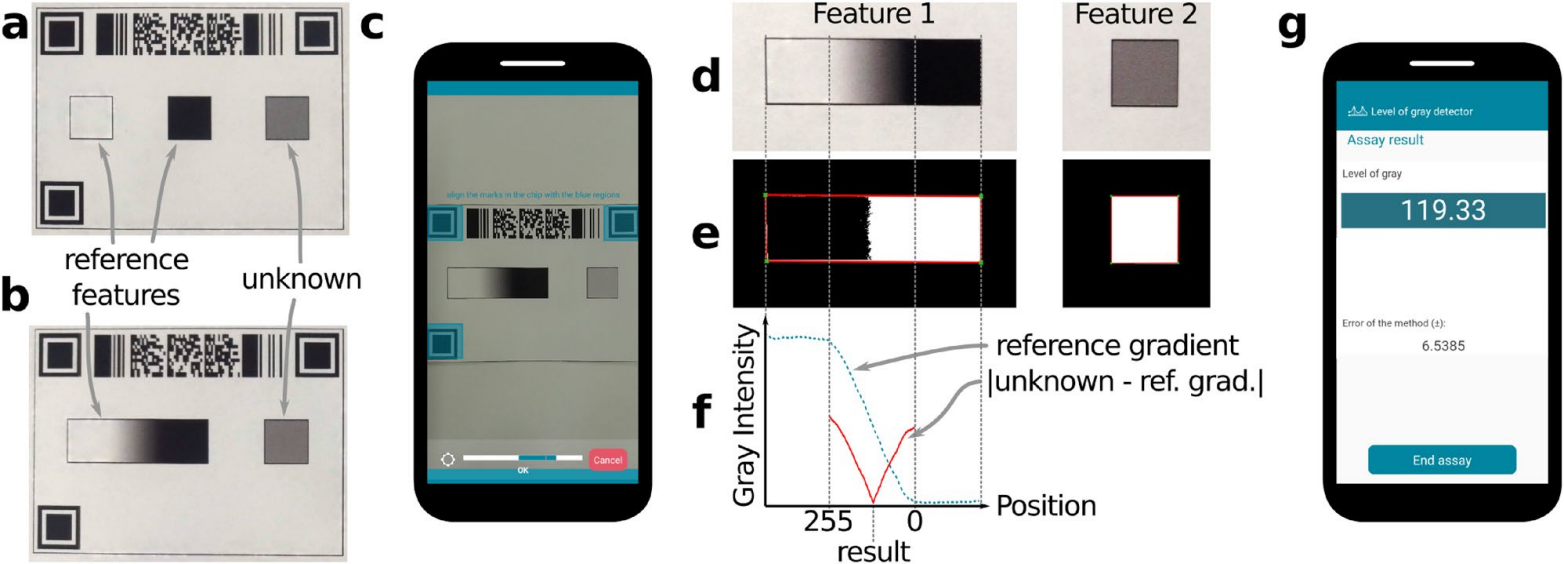
Application case #1: grayscale detector. The chips used include two kinds of features: the unknown and the color references, provided as (**a**) the extreme gray values or (**b**) a gradient with the full palette. (**c**) A screenshot of the mobile app showing the auto-shooting step. (**d**) The cropped features are passed to the algorithm for processing. (**e**) The algorithm segments the analyzed features in order to recognize the pixels containing useful information. (**f**) Plot of the measured reference gradient and the absolute error (between such gradient and the mean unknown value) as a function of position. The position where the error reaches a minimum is proportional to the unknown gray value. (**g**) Screenshot of the final report showing the obtained result.

The developed platform was tested with a simple application case meant to measure the level of gray of a region within the chip, mimicking tests based on colorimetric reading, where the amount of analyte can be correlated to the intensity of the reflected light. On the other hand, this application case also allows thorough characterization of the *appuente* performance, and serves as a tutorial from which new developers can start their projects. The chips used are depicted in Fig. 3a,b, where the alignment marks, the ID code and the features (color references and the unknown level of gray) can be observed. Following the flow diagram for developers in Fig. 2, a new project was created and general information about the test was filled in, as well as geometric information about the chip, feature sizes and positions. Next, an Octave algorithm (described in *Methods*) was selected after being coded and debugged in the desktop version of GNU Octave. The result returned by this algorithm is informed on a custom report shown on the smartphone screen (Fig. 3g). Next, the chips were printed on regular copy paper and cut. Proper functioning of the whole project was achieved after a couple of iterations of the previously described workflow.

In order to quantify the performance of the platform, several studies were carried out, whose results are collected in Table 1. The first five rows measure the error in determining a known level of gray under different situations. In particular, the fist two rows compare the performance of two different ways of providing the color references: the simplest one, where the extreme white and black references are provided (Fig. 3a) and an enhanced version, where the complete gray gradient is printed on the chip (Fig. 3b). In the third row, results obtained under ambient light intensity varying from 150 to 10,000 lx are compared. The fourth and fifth rows assess the variations obtained using different phone models and by different operators. The last two rows measure the ability to find the actual feature within the chip, as a function of the feature size. Again, two alternatives are compared: the simplest one, using the native capabilities of the platform, and an enhanced version using image processing techniques to find the actual feature within the image, as shown in Fig. 3d,e. Finally, results obtained using Octave where compared with Matlab (vR2017a, mathworks.com). For this purpose, a subset of the images from the experiment in the second row of Table 1, were analyzed with the same algorithm used for Octave, with minimum differences due to sintaxis variation between both programs. Results obtained were practically equivalent (differences lower than 0.1%).

**Table 1:** Performance of *appuente*. Percent error = $100|g_o-g_e|/255$, where $g_e$ and $g_o$ are the expected and the obtained level of gray, respectively. $A_c$ = area of the desired feature effectively captured, $A_p$ = real area of the feature. Ranges represent the minimum and maximum values obtained after five runs (n = 5).

Color determination percent error (simple alg.) vs. level of gray	0 0.7 ± 0.1%	64 8.3 ± 0.2%	127 5.6 ± 0.3%	191 2.5 ± 0.4%	255 4.6 ± 0.7
Color determination percent error (enhanced alg.) vs. level of gray	0 2.0 ± 0.7%	64 1.0 ± 1.4%	127 1.5 ± 0.7%	191 3.3 ± 1.5%	255 3.2 ± 2.4
Color determination percent error vs. ambient light intensity	150 lx 3.3 ± 1.6%	300 lx 1.4 ± 0.6%	1000 lx 4.1 ± 0.5%	3000 lx 1.7 ± 0.6%	10,000 lx 2.3 ± 0.6
Color determination percent error vs.smartphone model	TCL L10+ 1.2 ± 0.8%	Moto g6 plus 1.2 ± 1.3%	Redmi Note 8 1.7 ± 0.8%	Samsung J2 1.1 ± 1.3%	
Color determination percent error vs. operator	Operator 1 1.7 ± 0.7%	Operator 2 0.4 ± 1.0%	Operator 3 1.4 ± 1.3%	Operator 4 0.1 ± 1.0%	Operator 5 1.4 ± 0.6%
Native feature crop accuracy ($A_c/A_p$) vs. feature side	2.5 mm 37.8 ± 27%	5.0 mm 55.5 ± 22%	10 mm 66.5 ± 16%	15 mm 81.6 ± 7%	20 mm 82.9 ± 6%
Enhanced feature crop accuracy ($A_c/A_p$) vs. feature side	2.5 mm 91.4 ± 4%	5.0 mm 95.2 ± 2%	10 mm 96.9 ± 2%	15 mm 97.6 ± 1%	20 mm 98.7 ± 1%

### Application case #2: Device for root elongation assays

As mentioned in the Introduction, *appuente* was applied to a bioassay for measuring root elongation, which is referred to as Plant chip. Root elongation assays are commonly used to assess the phytotoxicity of chemical compounds. Seeds of certain species are grown and, after a specified time period, the root lengths of test and control groups are compared. This procedure is traditionally carried out in Petri dishes, which requires several time-consuming tasks. Plant chip was recently presented as a surpassing alternative, where roots are grown in an array of 20 milli-channels, allowing the operator to measure root lengths directly from a digital picture with the aid of image processing software^52^. The combination of Plant chip and *appuente* is proposed here to further improve and simplify the measurement procedure, as well as to evaluate root elongation as a function of time, which is a current need in the field^52^. Table 2 summarizes the different steps necessary for root elongation assays in different formats: the conventional assay on Petri dishes, Plant chip, and the combination of Plant chip and *appuente*.

**Figure 4:**
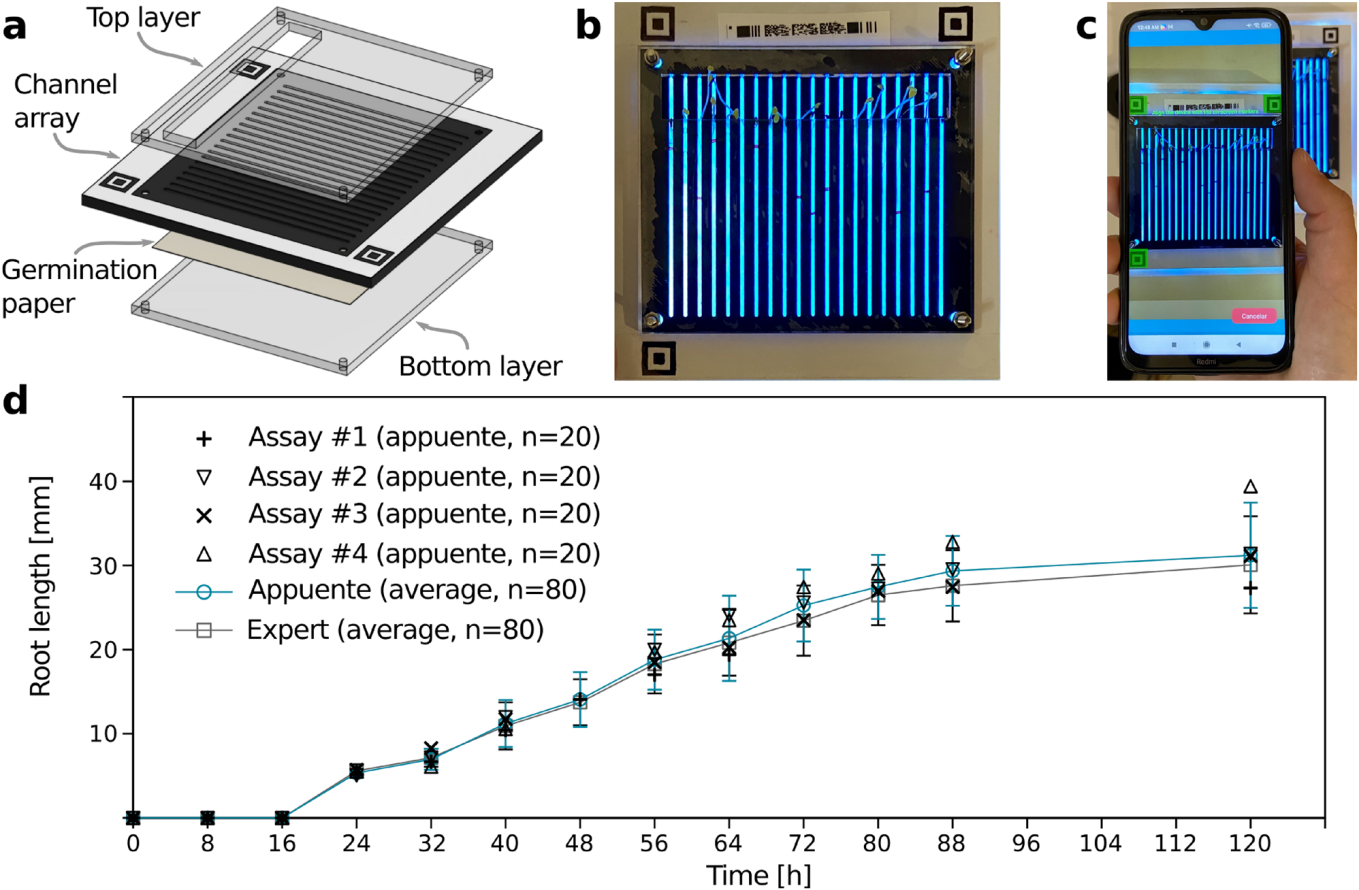
Application case #2. (**a**) Exploded-view drawing of the Plant chip device. (**b**) Photograph of the device showing an assay in progress and the red marks made during the reading process as prompted by the mobile app. (**c**) Automatic imaging of the chip by using the *appuente* app. (**d**) Root lengths as a function of time. Symbols ($+$ $\times$ $\bigtriangleup$ $\bigtriangledown$) represent the results returned by the app in four different experiments. In the same plot, the average values of the analyzed roots at different times (solid line-circle) are compared to the measurements made by an expert using the original Plant chip method (solid line-squares).

For this application case, the original Plant chip devices were modified to enhance contrast, using 3D-printed black PLA milli-channel array (Fig. 4a). A detailed description about plant chip fabrication can be found in the Methods section, as well as in the previous publication^52^. The *appuente* platform was used to produce a mobile app in which the user is prompted to (i) scan the ID code placed at the top of the chip, (ii) draw a red mark over each channel at the end position of every root (Fig. 4b), and (iii) align the chip for the app to automatically take a picture (Fig. 4c). Then, the cropped channel area is fed to the custom algorithm, the result of which is returned and shown in a final report that indicates the mean measured length and the standard deviation. An alternative procedure avoids the red mark step by using a more complex algorithm, which is capable of detecting roots and performing measurements directly. Such an approach makes operation even simpler, at the expense of additional processing time and the requirement of higher resolution images. To overcome these issues, a third alternative that makes use of machine learning techniques is currently being explored (not shown here). Figure 4d shows the results obtained using the simpler approach, in which the average root length obtained in four different experiments (n = 20, seeds per experiment) is shown as a function of time. In the same figure, the average length of the 80 analyzed roots is compared with the measurements performed by an expert, using the original Plant chip approach.

**Table 2:** Tasks required in each format of the root elongation assays: classical method on Petri dishes, original Plant chip device, and Plant chip combined with *appuente*. The listed tasks correspond to measurement and data gathering; the previous assay steps (seed positioning, working solutions, incubation) are common to all formats. In the simplest approach (see text for details) *appuente* requires manual pen marks.

Classical assay	Plant chip	Plant chip + appuente
Root removal	Picture	Run *appuente*
Root alignment	Image processing	
Manual measuring	Data processing	
Record values	Data storage	
Data processing		
Data storage		

### Application case #3: LFIA chips for leptospirosis detection

**Figure 5:**
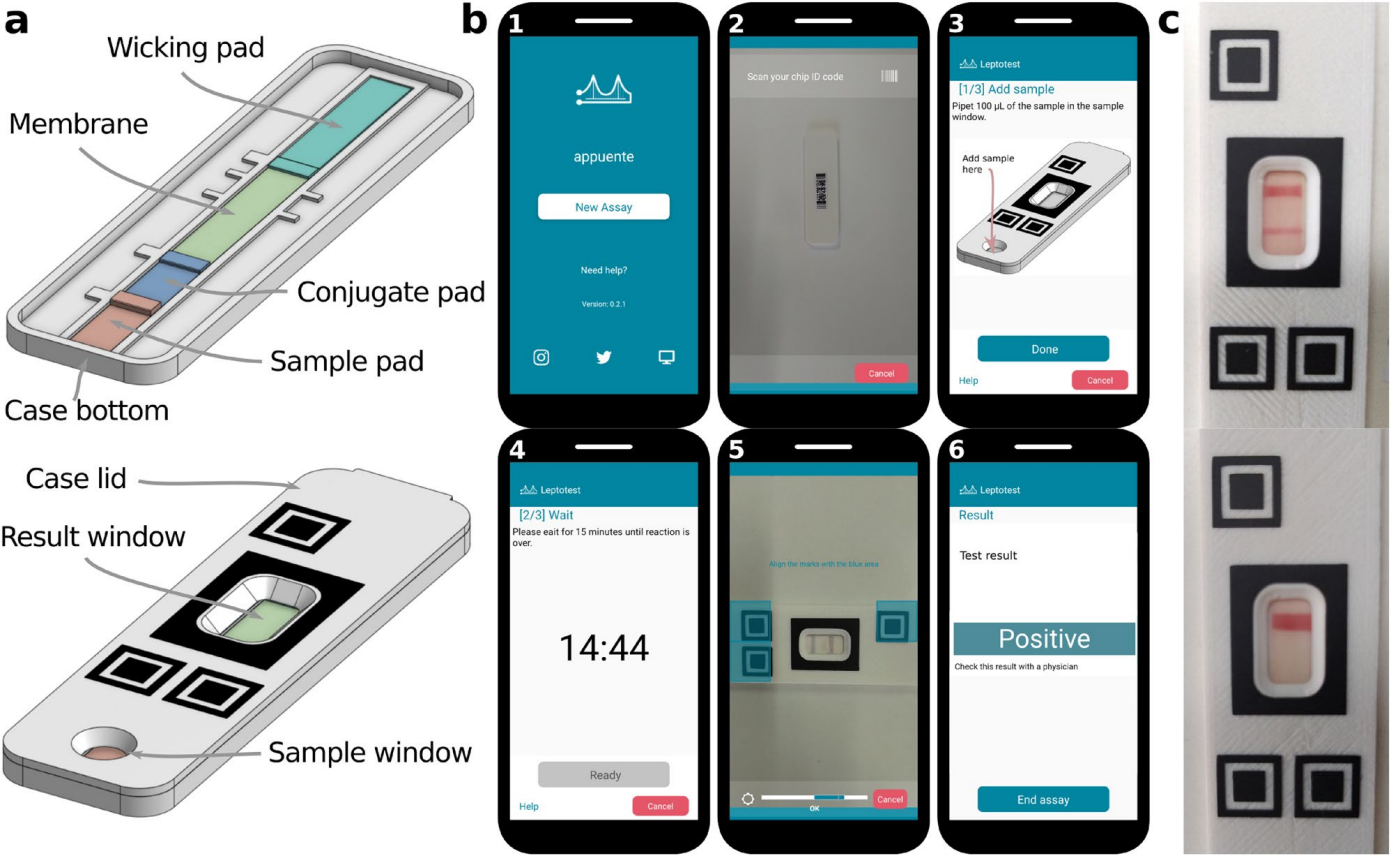
Application case #3. (**a**) A 3D render of the LFIA parts (top panel) and its cassette (bottom panel). (**b**) Screenshots of the custom mobile app created using *appuente*, at six running steps: (1) Start new test, (2) scan ID code, (3) add sample, (4) chronometer, (5) automatic imaging and (6) result report. (**c**) Real examples of LFIA chips for positive (top) and negative (bottom) samples.

Leptospirosis is a zoonotic infectious disease caused by pathogenic bacteria of the genus leptospira. Although leptospirosis is spread worldwide, it constitutes a special health problem in humid areas, such as tropical and subtropical regions, where most low-resource populations are found. Leptospirosis can be fatal, but effective and specific treatment is available, making early diagnostic valuable^56^. Recently, a LFIA for leptospirosis detection was developed^55^, which captures specific human IgM using a bacterial lysate as the antigen. The test showed high diagnostic performance, constituting a potential candidate for the implementation of early and accurate detection of leptospirosis. For this application case, the described LFIA strips were placed in custom 3D-printed plastic cassettes, with embedded alignment marks on top and individual ID codes at the bottom. The parts of the LFIA strip and the cassette are shown in Fig. 5a. The *appuente*  platform was used to produce the corresponding mobile app, capable of guiding and prompting the user through the required steps (Fig. 5b). The app also allows online reading of the assay results, by using a custom made algorithm capable of classifying test as “Positive”, “Negative” or “Invalid”. The result is indicated in a final report on the phone screen, avoiding user subjectivity, specially relevant in cases where the intensity of test lines is weak. The performance of the *appuente* enhanced LFIA for leptospirosis was evaluated with four different analyte dilutions (n = 5). Results were read by an experienced operator using the naked eye and, immediately after, by using the mobile app running on two different smartphones. The classification effectiveness was 100%, i.e., the expert and the mobile app on each cellphone correctly identified all positive and negative cases. Moreover, the images produced by the mobile app were saved and shown to a panel of ten non-expert operators, who were asked to classify the test results. The average classification effectiveness was 79.5% (min 55% , max 90%). Also, the inter-observer agreement, measured with the Fleiss’s $\kappa$ coefficient^57^ was 0.63, indicating intermediate agreement among the members of the panel. Examples of prototypes used for the experimental runs are shown in Fig. 5c.

## Discussion

The developed platform was successfully implemented and validated with three application cases, which in turn produced valuable information about the platform performance. Besides, it was evident how *appuente* improved several aspects of the assays, as described next. The grayscale detector showed a good performance in the color determination accuracy (percent error below 10%), with a simple algorithm that assumes a linear trend between black and white color references. As expected, the alternative that includes the full gray intensity palette for direct comparison with the unknown showed better performance. Even better performance should be possible after solving some remaining issues (regarding perspective and accurate indication of the beginning and end positions of the gray gradient) and allowing for a non-linear gradient to provide extra sensitivity. Thus, the grayscale detector showed that the color determination percent error can be very low (below 5% in all cases and around 1% in most cases), provided that proper algorithms and color references for calibration are used. Moreover, outstanding reproducibility was obtained, with error ranges below 5% in every case, and below 2% in most cases. Results obtained with this configuration were found to be independent from the operator, phone model, algorithm codification language, and light intensity. In the latter case, it should be noted that auto-shooting was not possible below 150 lx. Concerning cropping accuracy, native capabilities admit improvement, but they can also be significantly enhanced using image segmentation algorithms (Fig. 3e), as reported in Table 1.

Regarding the root elongation assay, it is readily observed in Table 2 how *appuente* reduces the number of experimental steps. Correspondingly, there is a clear decrease in assay complexity, time consumption, and the possibility of operator errors. As it can be seen in Fig. 4, the method showed very good agreement with the original Plant chip method considering both averages and standard deviations, with the absolute relative error ($<{10}$% in every case) being significantly smaller than the typical standard deviation of the assay. Thus, the approach proposed here is as accurate an reproducible as the validated assay formats, although significantly simpler and less time demanding. Furthermore, the combination of Plant chip and *appuente* allows for running time-dependent assays (Fig. 4d), which are time-consuming with Plant chip alone, and impossible to implement on traditional Petri dishes (where root elongation measurements can only be made at the end of the incubation). Live imaging provides rich information about the biological events that occur during plant development, particularly germination and root elongation, which can now be successfully followed without interrupting the experiment.

The platform was also tested with a POCT application, by implementing a novel LFIA for leptospirosis detection. Most of the platform capabilities were used: from the “no re-use” control, through user guidance, to smart-imaging of the chips. The performance of the mobile app in correctly classifying all the samples was compared against an expert, having both 100% effectiveness. When compared to users with no specific knowledge on LFIA tests, the average effectiveness was < 80% and the inter-operator agreement $\kappa$ was 0.63, indicating intermediate agreement among the operators. This means that the user-independent reading method presented here, clearly contributes improving result interpretation, even when naked-eye reading is possible, given that the user subjectivity plays a significant role for the assay outcome.

It should be noted that not every qualitative LFIA test might need integration with *appuente* or mobile devices in general. Examples are test not prone to weak positives, or those where false negatives do not constitute a risk. In such cases, naked-eye qualitative reading might be enough. It should be stated though, that the other advantages listed in the introduction can still be found of value, and justify integration with *appuente*.

The developed platform allows for fast prototyping of mobile apps for the integration with IoT and microfluidic devices. It was conceived to provide all the advantages offered by mobile phones: user guidance, chip identification and tracking, user control, processing, report, cloud and IoT integration and imaging. In particular, *appuente* provides smart-imaging capabilities, in which the geometric variability is minimized. The platform is also prepared to deal with other sources of variability, like camera model and settings and ambient light intensity and color. Specifically, it can be achieved by providing proper references in the chip, as shown in Fig. 3a,b. The aforementioned elements allow not only qualitative determinations, like in application case #3, but also quantitative assessments, as demonstrated in application cases #1 and #2.

Future work will focus on adding iPhone compatibility to the platform, data analytics, and offline operation. Native cropping capabilities will also be improved (a workaround was presented in this article). Some challenging issues still remain within the field, namely: hygiene and cross-contamination (particularly when human samples are involved), phone anchoring, and the handling of light non-uniformity, especially for applications using smartphones without additional hardware. Concerning hygiene, it will depend on the specific biological marker under detection, as well as if the *user* is also the *patient*, so that several ad hoc solutions could be explored. Regarding light non-uniformity handling, video based approaches have been proposed^58^. Also, using the phone LED light has been proposed, although such approach fails in situations where ambient light sources are prevalent. Another alternative requires locating the chip between the dominant light source and the user^41^, although it is highly user-dependent. The last two approaches can be addressed by *appuente*, by automatically turning on the LED light when shooting and/or by providing additional guidance to the user. Moreover, specific features can be positioned within the chip to measure light gradients and correct or discard the result if uniformity is not proper. Finally, even if several aspect are to be improved, the here presented free, flexible and customizable tool for the integration of microfluidics and smartphones, will hopefully allow innovations and spreading of PONT-IoT applications.

## Methods

### Web and mobile apps

The web server runs on a LNMP stack (i.e. Linux, Nginx, MySQL, PHP), using an Ubuntu virtual machine (v20.04 LTS, ubuntu.com), a Nginx web server (v1.18.0, nginx.org), a MySQL database (v8, mysql.com), and PHP programming language (v8.0). GNU Octave (v5.2.0, gnu.org/software/octave) installed on the server also interacts with the rest of the elements. The website was developed using the Laravel framework (v8.12, laravel.com) for PHP, providing web services to connect with the mobile app using the JSON (json.org) format. The TCPDF library (tcpdf.org) is used to generate the ID codes. The web app requires no installation since it runs on a web browser, and can be accessed from appuente.com. Although this app runs ideally on any browser, it was mainly tested on Firefox (v88.0 mozilla.org) Chrome (v88.0.4324.96, google.com/chrome) and Safari (v15.0 apple.com/safari/). The mobile app was developed using the React Native framework (reactnative.dev), using JavaScript. An Android native module was developed in Java for image processing using the library openCV (v3.10, opencv.org). The web services send to and receive from the server the required information at every step. The app (appuente 1.0.0) can be downloaded from the Google Play Android store and from appuente.com. For both the web and mobile apps, GitLab (gitlab.com) was used for version control.

### Grayscale detection chips and project setting

For the grayscale detector application example, two *appuente* projects were created, one for the chip shown in Fig. 3a, and the other for the chip in Fig. 3b. In both cases, a single smart-imaging step (Fig. 3c) was used. The “geometry and features” sections were filled with the desired physical dimensions of the chips, and proper algorithms were chosen. In the first case, containing three features, the algorithm (grayScaleDetect3.m, see Supplementary Information) averages the RGB pixels at each one of the features, converts it to the grayscale, and interpolates between the white reference (considered as 255) and the black reference (considered as 0) to find the level of gray of the unknown. In the second case, including two features, the algorithm (grayScaleDetect4.m, see Supplementary Information) converts the RGB values to grayscale, and finds the best match of the unknown within the reference gradient, containing all the possible gray values (Fig. 3f). In both cases, the features as defined in *appuente* were considerably larger than their physical counterparts, to make sure that no information is lost during the cropping procedure. Therefore, as a first step, the mentioned algorithms need to search for the *physical* feature, which is achieved using image segmentation. The chip geometry, with the embedded align marks and ID codes, was downloaded from the “Batch management” tab of the project and edited in Inkscape (v1.1, inkscape.org). Then, the chips were wax-printed (Xerox ColorQube 8580, Norwal, USA) on regular copy paper.

### Grayscale detection experiments

The described grayscale detector was used in different sets of experiments keeping the following conditions constant, unless noted: ambient light intensity = 300 lx, feature side = 10 mm, unknown level of gray = 127, smartphone brand-model = TCL L10+, algorithm = grayScaleDetect4.m. In the two first sets of experiments (Table 1), the accuracy achieved when measuring different levels of gray was studied when grayScaleDetect3.m and grayScaleDetect4.m were used, respectively. Next, the accuracy obtained under variable ambient light intensity (measured with the ambient light sensor embedded in the same phone) was determined. After that, the accuracy obtained using different smartphone models and operators was assessed. Finally, the feature cropping accuracy was evaluated when the native capabilities of the platform were used, and when those capabilities were enhanced using the image segmentation algorithms previously mentioned. In every case, 5 repetitions were performed for each experiment (n = 5). In all the experiments, the only measure taken for light uniformity, was to place the chips between the light source and the operator to avoid shadowing^41^.

### Root elongation chip preparation and project setting

For this application example, laser-cut PMMA top and bottom layers were used, as in the original device^52^. These transparent layers serve as the top and bottom walls of the channel where the roots grow, while allowing direct optic measurements. The middle layer was replaced by a black 3D-printed channel array, in order to enhance contrast and improve the performance of automatic identification and subsequent data analysis from (possibly low-resolution) images. The ID codes, obtained from the *appuente* environment, were printed on copy paper and glued to the chip. Germination paper was used as a substrate for the seeds (*Lactuca sativa*), the growing roots, and for water supply^52^.

After creating a new *appuente* project, the general data and chip geometry were set. Then, a custom algorithm was created and validated in a desktop version of GNU Octave. The algorithm (plantchip.m, see Supplementary Information) starts by identifying the locations of each of the 20 channels in the device, after which it looks for the relative position of the mark (or lack thereof) on each channel to output the corresponding physical root lengths. Algorithmic identification of the channels allows proper data analysis even in the presence of minor distortions in the image (as may be introduced by the smartphone’s camera lens), which can be expected due to the wide device compatibility of the *appuente* platform.

### Root elongation chip experiments

Germination paper was first moistened with DI water (2.55 mL) and a seed was placed in every channel. Incubation temperature was kept at 24 ± ${2}^\circ {\hbox {C}}$. Assays were performed in fourfold (n = 80) for 120 h each. When possible, images were taken every 8 h using the mobile app. Illumination was achieved by using a 12 W LED panel as a backlight, attenuated with a thin, light blue cardboard sheet. The *appuente* platform determined the root lengths as described above. For comparison, the images captured by *appuente* were also used by an expert to measure the root lengths with the *straight line* tool from ImageJ (v1.53k, imagej.nih.gov/ij/). The global average and standard deviation were computed from both the app’s and the expert’s measurements. In all cases, channels with no detectable ($< {5}\,{\hbox {mm}}$) or inconsistent growth were removed from the analysis.

### Leptospirosis LFIA chip preparation and *appuente* project setting

Plastic cassettes were 3D printed on PLA and the alignment marks were cut in self-adhesive vinyl. ID codes, obtained from the *appuente* environment, were printed on copy paper and glued to the case bottom. LFIA pads were assembled manually into the plastic cassettes, using the guides 3D printed at the bottom. Pads were mounted on two-sided tape in the following order: reaction pad (FF120HP, GE Life Sciences), conjugate pad (Fusion 5, GE Life Sciences) containing a solution of gold nanoparticles conjugated to goat anti-human IgM, sample and absorbent pads (CF3, Whatman, GE Life Sciences). Then, test and control lines were drawn onto the reaction pad by dipping silicone seals, respectively, into the leptospiral antigen preparation or a rabbit anti-goat IgG solution (Sigma-Aldrich) and stamping on the nitrocellulose membrane according to demarcated positions on the cassettes.

An *appuente* project was created and properly configured to guide the user through the required steps: sample load, 15-min incubation, and reading (see Fig. 5b). Geometric properties were also loaded on the project to match the cassette marks. A single feature was defined coincident with the black rectangle around the result window. An Octave algorithm (lfiaRead3.m, see Supplementary Information) was created, which (i) automatically finds the result window within the feature area, (ii) converts the RGB to a gray scale signal and takes average values in the longitudinal direction of the window, (iii) obtains a binary signal using an experimentally predefined threshold, (iv) looks for coincidences between the binary signal and the expected positions for the test and control lines, (v) uses this information to classify the test as positive, negative, or invalid.

### Leptospirosis LFIA chip experiments

The chips for leptospirosis detection were tested with two real serum samples corresponding to a negative and a positive case of leptospirosis, classified at *Laboratorio Nacional de Referencia de Leptospirosis* of the *Instituto Nacional de Enfermedades Respiratorias (INER) “Dr E. Coni”*, Santa Fe, Argentina. Four different analyte concentrations were assessed in quintuplicate (n = 5): 1:100 dilution of the negative sample, 1:100, 1:200 and 1:500 dilutions of the positive sample. One hundred microliters of each were dispensed into the sample window of the chip and after 15 min, results were read and classified in two ways: naked eye reading by an expert and running the mobile app on different smartphones (TCL L10+ and Xiaomi Redmi 8). In addition, a non-expert panel was assembled to classify each sample using the images taken by *appuente*. The panel was composed of ten adults (ages: 27–39 y.o.) with degrees in natural sciences, who received a brief explanation of the task. The classification effectiveness of the expert, the app and the panel was calculated as the percentage of samples correctly classified (as negative, positive or invalid), compared to the known sample composition. The inter-operator agreement was measured using the Fleiss’s $\kappa$ coefficient^57^, as a measure of the degree of concord among the members of the panel, accounting for chance. Complete agreement among the members of the panel means $\kappa =1$ while agreement equal to the expected by chance, means $\kappa =0$. The LFIA assays were evaluated and approved by the Bioethics Committee of FBCB, UNL (CE2019-37, Acta 05/19). All the experiments were performed in accordance with relevant national and international guidelines^59,60^. Informed consent was obtained from all participants and/or their legal guardians to use their samples for the research.

## Supplementary Information

Supplementary Information.

Supplementary Video 1.

## Data Availability

The datasets analyzed in this article are available from the corresponding author on reasonable request. The GNU Octave algorithms used for this work are available in the Supplementary Information.
