# Supplementary material for: A free customizable tool for easy integration of microfluidics and smartphones
Source: Sci Rep. 2022 May 27;12:8969. doi: 10.1038/s41598-022-13099-z (PMC9142529; doi:10.1038/s41598-022-13099-z)
Supplement: Supplementary file 1 — Supplementary Information. [file 41598_2022_13099_MOESM1_ESM.pdf]

SUPPLEMENTARY INFORMATION - A free customizable tool for easy integration of microfluidics and smartphones

Federico Schaumburg[1,*], Juan P. Vidocevich[1], Gabriel S. Gerlero[2], Nazarena Pujato[3,+], Joana Macagno[1,+], Pablo A. Kler[2], and Claudio L. A. Berli[1,]**

[1]Instituto de Desarrollo Tecnológico para la Industria Química (INTEC, UNL–CONICET), Colectora RN 168 Km 472, Santa Fe, S3000GLN, Argentina
[2]Centro de Investigación de Métodos Computacionales (CIMEC, UNL-CONICET), Colectora RN 168 Km 472, Santa Fe, S3000GLN, Argentina
[3]Laboratorio de Tecnología Inmunológica (FBCB, UNL), Colectora RN 168 Km 472, Santa Fe, S3000GLN, Argentina
[*]fschaumburg@intec.unl.edu.ar
[**]cberli@santafe-conicet.gov.ar
[+]these authors contributed equally to this work

GNU Octave stuctures for appuente

The GNU Octave algorithm interacts with appuente through two data structures, one called *assay* and other called *report*. The former is filled by appuente and carries input information to be used by the algorithm. The last, must be filled in the .m file and carries the output from the algorithm to appuente. A scheme of this can be seen in Fig. 1 in the main text.

assay structure

The fields of the structure are described next.

`assay.general.name`: name of the test.
`assay.general.IDT`: code identifying the test.
`assay.general.IDB`: code identifying the batch.
`assay.general.IDC`: code identifying the chip.
`assay.general.dimensions.L`: length of the chip [mm].
`assay.general.dimensions.H`: height of the chip [mm].
`assay.general.dimensions.S`: align mark side [mm].
`assay.general.dimensions.C`: clearance between chip parts [mm].
`assay.general.t_initial`: initial time of the assay.
`assay.general.duration`: duration of the assay.
`assay.step(N).title`: title of the Nth step of the assay
`assay.step(N).detail`: detail of the Nth step of the assay
`assay.step(N).type`: type of the Nth step of the assay.
`assay.feature(N).name`: name of the Nth feature.
`assay.feature(N).description`: description of the Nth feature.
`assay.feature(N).length`: length of the Nth feature.
`assay.feature(N).height`: height of the Nth feature.
`assay.feature(N).x-coordinate`: horizontal position of the Nth feature within the chip.
`assay.feature(N).y-coordinate`: vertical position of the Nth feature within the chip.
`assay.feature(N).figure`: RGB picture of Nth feature within the chip, i.e. the cropped original image.

report structure

The fields of the structure are described next.

`report.result.value`: numeric result of the assay.
`report.result.value`: numeric result of the assay.
`report.result.error_plus`: superior error/uncertainty of the numeric assay result.
`report.result.error_minus`: inferior error/uncertainty of the numeric assay result.
`report.result.unit`: unit of the numeric assay result.
`report.result.text`: result of the test in a text format (e.g. "Positive/Negative").
`report.advice`: advice/disclaimer for the user regarding the result.
`report.figure.figure`: image related to the result (e.g. processed image of one feature of the chip).
`report.figure.caption`: text explaining the figure returned with `report.figure.figure`.

GNU Octave format of an algorithm for appuente

The typical format of the algorithm contained in the .m file is given next.

```
function report = algorithmName (assay)
  %load necessary packages
  pkg load image;

  \%access feature information
  I1 = assay.feature(1).figure;
  I2 = assay.feature(2).figure;

  %process images as desired

  %fill the report structure
  report.result.value = 12.0;
  report.result.error_plus = 1.0;
  %report.result.error_minus = 1.0;
  report.result.error_unit = "meters";
  report.result.error_text = "none";
  report.advice = "Please refer to a physician";

endfunction
```

GNU Octave algorithms used in this article

Next, the code of the algorithm used in the three application examples presented in this article are given:
-grayScaleDetect3.m
-grayScaleDetect4.m
-plantchip.m
-lfiaRead3.m

```
function report = grayScaleDetect3 (assay)

  tIni=time;

  %Necesary packages
  pkg load image;
  pkg load signal;
  pkg load statistics;

 %Feature physical dimmentions
  oS=18;
  margin=0.25;
  grayValues=[0 0 0];
  stdValues=[0 0 0];

  %Crop features
for ii = 1:length(assay.feature)
  I = assay.feature(ii).figure;

  %Image realted dimmentions
  Lenght = length(I(:,1,1));
  Width = length(I(1,:,1));
  areaDetection=0.85*(10**2/24**2)*Lenght*Width;
  Scale = (Lenght+Width)/oS/2;

  %Obtain binary image of the actual feature
  Ibw = im2bw(I,graythresh(I));
  Iarea = ~bwareaopen(Ibw,areaDetection,8);
  Ifinal = bwlabel(Iarea);
  reg = regionprops(Ifinal,'Area','ConvexHull','Extrema','BoundingBox');

 %If more than one region found, keep the bigest
  [nr,nc] = size(reg);
  if(nr>1)
    regArea = [reg(:).Area];
    [val idx] = max(regArea);
    reg = reg(idx);
  endif

  %Find feature corners among the ConvexHull points
  %Get the BoundingBox points
  bbaux = [reg(:).BoundingBox];
  %Top-left, top-right, bottom-right, bottom-left
  bB = [bbaux(1) bbaux(2); bbaux(1)+bbaux(3) bbaux(2); bbaux(1)+bbaux(3)
  bbaux(2)+bbaux(4); bbaux(1) bbaux(2)+bbaux(4)];
  %Choose the extrema as the points in ConvexHull more similar to the BoundingBox
  cH = [reg(:).ConvexHull];
  [vMin iMin] = min(pdist2(bB,cH),[],2);
  ext=cH(iMin(:),:);

  %Define poligon containig feature with a margin
  auxExt(1,:)=round(ext(1,:)+margin*Scale);
  auxExt(2,1)=round(ext(2,1)-margin*Scale);
  auxExt(2,2)=round(ext(2,2)+margin*Scale);
  auxExt(3,:)=round(ext(3,:)-margin*Scale);
  auxExt(4,1)=round(ext(4,1)+margin*Scale);
  auxExt(4,2)=round(ext(4,2)-margin*Scale);

  %Convert to gray scale and crop using a binary mask
  mask = poly2mask(auxExt(:,1),auxExt(:,2),Lenght,Width);
  cI=rgb2gray(I);
  cI=cI(mask);

  %Calculate std and mean gray values
  grayValues(ii)= mean2(cI);
  stdValues(ii) = std2(cI);
endfor

  %Fill White, Black and Unknown mean values and standard deviation value.
```

```
69      whiteMeanValue=grayValues(1);
70      blackMeanValue=grayValues(2);
71      unknownMeanValue=grayValues(3);
72      uStdValue=stdValues(3);
73
74      %The mean output value is computed
75      out = 255/(whiteMeanValue-blackMeanValue)*(unknownMeanValue-blackMeanValue);
76
77      %The corresponding unknown+std value is computed
78      e_M =
        255/(whiteMeanValue-blackMeanValue)*((unknownMeanValue+uStdValue)-blackMeanValue)-out;
79
80      %The corresponding unknown-std value is computed
81        e_m =
          out-255/(whiteMeanValue-blackMeanValue)*((unknownMeanValue-uStdValue)-blackMeanValue)
          ;
82
83      %Elapsed time
84      elapsed=time-tIni;
85
86      %The report structure is filled
87      report.result.value = out;
88      report.result.error_plus = max(e_M,e_m);
89      %report.result.error_minus = out_m;
90      report.result.error_unit = "";
91      report.result.error_text = "";
92      report.advice = cstrcat("Processing time [s]: ",num2str(elapsed));
93
94    endfunction
95
```

```
function report = grayScaleDetect4 (assay)

  tIni=time;

  %Necesary packages
  pkg load image;
  pkg load signal;
  pkg load statistics;


 %Feature physical dimmentions
  oL=24; %original feature length (it is actually the height)
  margin=0.5;
  gradIni = 0.29907; %Percent value where gradient starts within the feature
  gradEnd = 0.70093; %Percent value where gradient ends within the feature
  %Crop features
for ii = 1:length(assay.feature)
  I = assay.feature(ii).figure;

  %Image realted dimmentions
  Lenght = length(I(:,1,1));
  Width = length(I(1,:,1));
  areaDetection=0.85*(10**2/24**2)*Lenght*Width;
  Scale = Lenght/oL;

  %Obtain binary image & detect window region
  Ibw = im2bw(I,graythresh(I));
  Iarea = ~bwareaopen(Ibw,areaDetection,8);
  Ifinal = bwlabel(Iarea);
  reg = regionprops(Ifinal,'Area','ConvexHull','Extrema','BoundingBox');

 %If more than one region found, keep the bigest
  [nr,nc] = size(reg);
  if(nr>1)
    regArea = [reg(:).Area];
    [val idx] = max(regArea);
    reg = reg(idx);
  endif

  %Find feature corners among the ConvexHull points
  %Get the BoundingBox points
  bbaux = [reg(:).BoundingBox];
  %Top-left, top-right, bottom-right, bottom-left
  bB = [bbaux(1) bbaux(2); bbaux(1)+bbaux(3) bbaux(2); bbaux(1)+bbaux(3)
  bbaux(2)+bbaux(4); bbaux(1) bbaux(2)+bbaux(4)];
  %Choose the extrema as the points in ConvexHull more similar to the CoundingBox
  cH = [reg(:).ConvexHull];
  [vMin iMin] = min(pdist2(bB,cH),[],2);
  ext=cH(iMin(:),:);

  %Define poligon containig feature with a margin
  auxExt(1,:)=round(ext(1,:)+margin*Scale);
  auxExt(2,1)=round(ext(2,1)-margin*Scale);
  auxExt(2,2)=round(ext(2,2)+margin*Scale);
  auxExt(3,:)=round(ext(3,:)-margin*Scale);
  auxExt(4,1)=round(ext(4,1)+margin*Scale);
  auxExt(4,2)=round(ext(4,2)-margin*Scale);

  %Convert to gray scale and crop using a binary mask
  cI=rgb2gray(I(auxExt(1,2):auxExt(3,2),auxExt(1,1):auxExt(3,1),:));

  %Calculate std and mean gray values
  if(ii==1)
    refGradMean = mean(cI);
    refGradStd  = std(cI);
  endif
  if(ii==2)
    unknownMean = mean2(cI);
    unknownStd = std2(cI);
```

```
69      endif
70    endfor
71
72      %Obtain valuable part of gradient
73
        vRefGradMean=refGradMean(floor(gradIni*length(refGradMean)):ceil(gradEnd*length(refGrad
        Mean)));
74      %Search for the best match for the unknown within the gradient
75      [gMinVal gMinIdx] = min(abs(vRefGradMean-unknownMean));
76      out = 255*(1-gMinIdx/length(vRefGradMean));
77
78      %Search for the best match for the unknown+std within the gradient
79      [gMinVal gMinIdx] = min(abs(vRefGradMean-(unknownMean+unknownStd)));
80      out_M = 255*(1-gMinIdx/length(vRefGradMean));
81
82      %Search for the best match for the unknown+std within the gradient
83      [gMinVal gMinIdx] = min(abs(vRefGradMean-(unknownMean-unknownStd)));
84      out_m = 255*(1-gMinIdx/length(vRefGradMean));
85
86
87      %Elapsed time
88      elapsed=time-tIni;
89
90      %The report structure is filled
91      report.result.value = out;
92      report.result.error_plus = max(out_M-out, out-out_m);
93      %report.result.error_minus = u_m;
94      report.result.error_unit = "";
95      report.result.error_text = "";
96      report.advice = cstrcat("Processing time [s]: ",num2str(elapsed));
97
98    endfunction
99
```

```
function report = plantchip(assay)

  pkg load image;
  pkg load statistics;

  %--- SETUP ---%

  IDT = 14;
  NCHANNELS = 20;
  CHANNEL_AREA_LENGTH = 63.0;

  assert(assay.general.IDT, IDT);

  img = assay.feature(2).figure;
  pixel_density = size(img, 1)/CHANNEL_AREA_LENGTH;

  %--- CHANNEL DETECTION ---%

  threshold = img(:,:,2) > 125;
  dthreshold = diff(threshold, 1, 2);

  edge_pos = NaN(2*NCHANNELS, size(img, 1));

  for j=1:size(edge_pos, 2)
    indices = find(dthreshold(j,:));
    if (length(indices) == 2*NCHANNELS)
      edge_pos(:,j) = indices;
    end
  end

  assert(size(edge_pos, 1), 2*NCHANNELS);
  assert(size(edge_pos, 2), size(img, 1));

  if (all(isnan(edge_pos(1,:))))
    report.result.value = NaN;
    report.result.error_plus = NaN;
    report.result.error_minus = NaN;
    report.result.unit = "mm";
    report.result.text = "Failed to identify all root channels in the image";

    report.advice = "Please try taking a new photo of the device";
    return
  end

  edges = zeros(2*NCHANNELS, 2);

  for i=1:size(edge_pos, 1)
    edge = edge_pos(i,:);
    valid = ~isnan(edge);
    result = polyfit(find(valid), edge(valid), 1);
    slope = result(1);
    intercept = result(2);
    edges(i,1) = intercept;
    edges(i,2) = intercept + slope*length(edge);
  end

  %--- MEASUREMENTS ---%

  MIN_ROOT_LENGTH = 5.0;

  lengths = NaN(NCHANNELS, 1);

  for c=1:NCHANNELS

    top_left = edges(2*c-1,1);
    top_right = edges(2*c,1);
    bottom_left = edges(2*c-1,2);
    bottom_right = edges(2*c,2);

```

```
    height_px = size(img, 1);

    length_px = norm([0, mean([top_left, top_right])] - [height_px, mean([bottom_left,
    bottom_right])]);
    pixel_density = length_px/CHANNEL_AREA_LENGTH;

    mask = poly2mask([top_left, top_right, bottom_right, bottom_left],
                     [0, 0, height_px, height_px],
                     size(img, 1),
                     size(img, 2));
    green = img(:,:,2);
    ignore_px = round(MIN_ROOT_LENGTH*pixel_density);

    line = NaN(size(green, 1)-ignore_px, 1);
    for i=1:length(line)
      l = green(ignore_px+i,:);
      m = mask(ignore_px+i,:);
      line(i) = mean(l(m)) <= 110;
    end

    mark = find(line);

    if length(mark) == 0
      continue;
    end

    length_px = ignore_px + mark(1) - 1;
    %length_px_max = ignore_px + mark(end) - 1;

    %assert(length_px_min <= length_px_max);

    %length_px = mean([length_px_min, length_px_max]);

    length_mm = length_px/pixel_density;

    if length_mm >= MIN_ROOT_LENGTH
      lengths(c) = length_mm;
    end
  end

  %--- RESULTS ---%

  avg = nanmean(lengths);
  stddev = nanstd(lengths);
  nroots = size(lengths, 1)-sum(isnan(lengths));

  %--- RETURN ---%

  report.result.value = avg;
  report.result.error_plus = stddev;
  report.result.error_minus = stddev;
  report.result.unit = "mm";
  report.result.text = strcat(num2str(nroots), " roots measured");

  report.plot.x_series = 1:size(lengths, 1)';
  report.plot.y_series = lengths;
  report.plot.x_error = 0*lengths;
  report.plot.y_error = lengths*nan;
  report.plot.x_title = "Channel";
  report.plot.y_title = "Root length [mm]";
  report.plot.caption = "Root lengths by channel";

  report.advice = "";
```

```
function report = lfiaRead3 (assay)

  %Necesary packages
  pkg load image;
  pkg load signal;

  I = assay.feature(1).figure;

  %Original dimmentions
  oL=17;
  oW=12;
  margin=1.8+0.5;

  %Image dimmentions
  iLenght = length(I(:,1,1));
  iWidth = length(I(1,:,1));
  areaDetection=0.5*iLenght*iWidth;
  scale = (iLenght/oL+iWidth/oW)/2;

  %Obtain binary image & detect window region
  Ibw = im2bw(I,graythresh(I));
  Iarea = bwareaopen(Ibw,areaDetection);
  Ifinal = bwlabel(Iarea);
  bbox = regionprops(Ifinal,'boundingbox');

  %Crop NC strip from window region
  bb = bbox(1).BoundingBox;
  auxL=round(bb(3)-2*margin*scale);
  auxH=round(bb(4)-2*margin*scale);
  auxX=round(bb(1)+margin*scale);
  auxY=round(bb(2)+margin*scale);
  strip = [auxX auxY auxL auxH];
  II=I(auxY:auxY+auxH,auxX:auxX+auxL,:);

  %The NC strip is converted to a gray value and averaged along y dimention. StdDev is
  also calculated
  grayMeanValue=mean(rgb2gray (II),1);
  gray3StdValue=1*std(rgb2gray (II));
  x=[1:length(grayMeanValue)];

  %Signal inverted and normalized. Outer parts removed.
  gMV_d=grayMeanValue;%detrend(grayMeanValue);
  gMV_d*=-1;
  gMV_din = (gMV_d-min(gMV_d))/(max(gMV_d)-min(gMV_d));
  gSTD = gray3StdValue/(max(gMV_d)-min(gMV_d));
  gMV_din(1:floor( 1.35*length(x)/10))=0;
  gMV_din(ceil(7.65*length(x)/10):end)=0;

  %Binary signal obtained using treshold obtained experimentally.
  mean_gMV=mean(gMV_din)*1.2;
  binSignal=gMV_din>=mean_gMV;
  binSignal+=0.0;

  %Masks for Test, Background and Control signal possible positions
  maskTest=zeros(1,length(x));
  maskTest(floor( 1.35*length(x)/10):ceil(3.15*length(x)/10))=1;
  maskBack=zeros(1,length(x));
  maskBack(ceil(4.0*length(x)/10):floor(4.8*length(x)/10))=1;
  maskControl=zeros(1,length(x));
  maskControl(floor(5.85*length(x)/10):ceil(7.65*length(x)/10))=1;

  %Dot product calculated to find coincidences
  positiveTest=(dot(maskTest,binSignal))>0;
  negativeBack=(dot(maskBack,binSignal))<=0;
  positiveCont=(dot(maskControl,binSignal))>0;

  if (positiveCont&&negativeBack)
    if (positiveTest)
        texto = "Positive";
```

```
69        else
70          texto = "Negative";
71        endif
72      else
73       texto = "Invalid ";
74      endif
75
76
77      %The report structure is filled
78      %report.result.value =;
79      %report.result.error_plus =;
80      %report.result.error_minus =;
81      %report.result.unit = "";
82      report.result.text = texto;
83      report.advice = "Check this result with a physician";
84
85    endfunction
86
87
```
